# Wide-field angiography in retinal vein occlusions

**DOI:** 10.1186/s40942-019-0163-1

**Published:** 2019-12-12

**Authors:** Colin S. Tan, Kelvin Z. Li, SriniVas R. Sadda

**Affiliations:** 1 National Healthcare Group Eye Institute, Tan Tock Seng Hospital, Jalan Tan Tock Seng, 11, Singapore, 308433 Singapore; 2 Fundus Image Reading Center, National Healthcare Group Eye Institute, Singapore, Singapore; 3 Duke-NUS Medical School, Singapore, Singapore; 4 Lee Kong Chian School of Medicine, Nanyang Technological University, Singapore, Singapore; 5 Doheny Eye Institute, University of California, Los Angeles, CA USA

**Keywords:** Retinal vein occlusion, Ultrawidefield imaging, Fluorescein angiography, Ischemic index, Macular edema

## Abstract

**Background:**

Retinal vein occlusion (RVO) is the second most common retinal vascular disease after diabetic retinopathy. It can result in significant visual loss from complications like macula edema, retinal and iris neovascularization, and vitreous hemorrhage. Recently, ultra-widefield imaging (UWF) has been developed for posterior pole visualization and has shown to be useful in the evaluation and treatment of RVO.

**Main text:**

Ultra-widefield imaging (UWF) imaging allows for visualization of the retina up to an angle of 200°. This is especially important in detecting peripheral retinal pathologies, especially in retinal conditions such as RVO, where the disease process affects the peripheral as well as central retina. In particular, retinal non-perfusion in RVO is a risk factor for neovascularization. Various techniques, such as ischemic index and stereographic projection, have been described to assess areas of ischemia on UWF images. Retinal non-perfusion has an impact on disease complications, such as macular edema, and retinal and iris neovascularization. Retinal non-perfusion also has implications on disease response, including visual acuity, reduction in retinal edema and treatment burden.

**Conclusion:**

Ultra-widefield imaging (UWF) imaging plays an important role in the assessment and management of RVO, especially in measuring retinal non-perfusion in the peripheries.

## Background

Retinal vein occlusion (RVO) is the second most common type of retinal vascular disease, after diabetic retinopathy [1]. A meta-analysis of 15 studies from the United States, Europe, Asia and Australia reported a prevalence of 4.42 per 1000 for central RVO (CRVO) and 0.8 per 1000 for branch RVO (BRVO) [2]. The Beaver Dam Eye Study reported a prevalence of 0.6% for BRVO, and 0.1% for CRVO [3]. The prevalence of RVO increases with age. In the Beaver Dam Eye Study, persons aged 75 years or older were 6.7 times more likely to have BRVO compared to those aged between 43 and 54 years [3].

Major risk factors for RVO include hypertension [2, 4, 5], arteriosclerosis, hyper-triglyceridemia [4] and glaucoma [2, 6]. In young patients, however, CRVO is more commonly associated with hematological abnormalities or pro-coagulant conditions, such as anemia, polycythemia, leukemia, multiple myeloma, abnormal platelet function and reduced anti-thrombin III [7].

RVO may result in significant visual loss. In the Central Vein Occlusion Study (CVOS) [8], visual outcomes varied according to baseline visual acuity (VA). Among those with good initial VA (defined as VA 20/40 or better), 65% maintained VA in the same range for the duration of the study. Patients with intermediate VA at baseline (20/50 to 20/200) had variable outcomes, with 19% showing improvement in VA, 44% remaining in the same range, and 37% worsening by the end of the study. In contrast, those with poor VA at baseline (worse than 20/200) had an 80% chance of remaining worse than 20/200.

### Pathophysiology of RVO

Patients with RVO may experience visual loss from macular edema or vitreous hemorrhage and the complications related to this.

RVO is believed to result from compression of the retinal vein by the corresponding retinal arteriole, which is stiffened as a result of underlying hypertension and arteriosclerosis. This, together with damage to the vessel wall, results in thrombus formation [9]. Vascular occlusion leads to an increase in intraluminal venous pressure, which subsequently results in capillary endothelial cell damage, retinal hemorrhages, and eventually capillary dropout [10]. Capillary dropout and hypoxia then cause inflammation and an up-regulation of pro-inflammatory cytokines, such as vascular endothelial growth factor (VEGF) [11].

Increased VEGF production causes local inflammation and increased vascular permeability, which results in macular edema [11]. Studies have shown that the levels of both stimulatory cytokines, such as VEGF, and inhibitory cytokines like pigment epithelium-derived factor (PEDF), are correlated with the severity of macular edema [12, 13].

Retinal ischemia resulting from RVO may result in neovascularization, either in the anterior segment or in the retina. Neovascularization of the iris or angles may progress to neovascular glaucoma, while retinal neovascularization may bleed, resulting in vitreous hemorrhage [2, 14].

## Importance of ultra-widefield imaging in retinal vein occlusion

Imaging plays an increasingly important role in ophthalmology [15–22], particularly for retinal conditions. Historically, flash color fundus photographs were obtained using 35-mm slides, which provided a 30° field of view after pupil dilation. This field of view allowed visualization of approximately 5% of the total retinal area [23].

Subsequent advances in fundus cameras and the advent of digital fundus photography allowed larger single fields of view to be obtained, ranging from 45° to 50°. The coverage of the retina was increased by steering the eye in different directions of gaze, and obtaining overlapping retinal photographs.

One common method that has been used in many clinical trials is the Early Treatment Diabetic Retinopathy Study (ETDRS) 7 standard field (7SF) [24]. Using this method, 7 overlapping stereographic photographs were taken and assessed. Together, these images covered a width of 75° or approximately 30% of the entire retinal surface [25].

More recently, ultra-widefield (UWF) imaging has been developed for posterior pole visualization [26]. Using devices such as the Optos 200 Tx or Optos California, images covering an angle of 200° can be obtained in a single image. This accounts for approximately 80% of the retinal surface [27]. By steering the eye in different directions of gaze, additional areas of the retina can be covered, and it is possible to visualize the ora serrata in cooperative patients [28].The steered images can also be montaged to obtain a single composite image. Ultra-widefield imaging has been used in color fundus photography [29], fundus autofluorescence [30–32], fluorescein angiography (FA) [21, 33–35] and indocyanine green angiography [36].

**Fig. 1:**
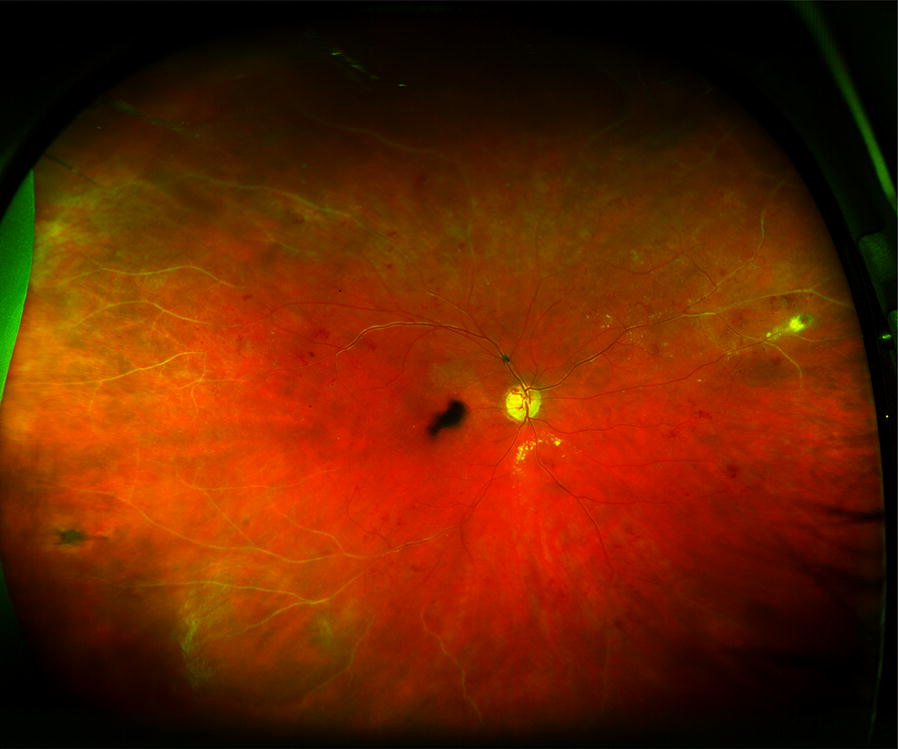
Ultrawidefield fundus photography of central retinal vein occlusion. The pathology extends beyond the region covered by standard fundus cameras. In particular, sclerosed vessels can be seen in the peripheral retina. Optic disc collaterals are seen

UWF images have several advantages compared to conventional fundus photography. In particular, a large portion of the retina may be captured on a single image (Fig. 1), whereas conventional fundus photography requires multiple images and still covers a smaller area of the retina. Thus UWF imaging may avoid the need to montage images, where artefacts may occur at the borders of the overlapping images, or lesions which occur at the regions of overlap may be masked [37].

In addition, UWF images can be obtained without pupil dilation, thus minimizing inconvenience to the patient. Acquiring UWF images is also faster compared to conventional photography. In one study, the time taken to acquire nonmydriatic UWF images was significantly shorter compared to that of dilated fundus photography in the ETDRS 7 standard fields (170 ± 80 vs. 370 ± 130 s, p < 0.001) [29].

Images taken using the Optos device also have high resolution (3900 × 3072 pixels, which allows 17 to 22 pixels per degree of view [38]. In addition, the Optos pseudocolor images are taken using different wavelengths, which allow visualization of the different layers of the retina using the red or green filters.

Studies have demonstrated the importance of detecting peripheral retinal pathology in various retinal conditions, including RVO [33, 39], diabetic retinopathy [37, 40, 41] and age-related macular degeneration [30, 31]. For example, in diabetic retinopathy, lesions may be located more peripherally, predominantly outside the ETDRS 7SF, and would be missed using conventional imaging [25].

### Importance of widefield angiography in RVO

The disease process in RVO affects the peripheral as well as central retina, and lesions may be located well beyond the posterior pole (Fig. 1). One of the sequelae of RVO is retinal non-perfusion, which is a risk factor for iris and retinal neovascularization [42, 43]. In addition, retinal non-perfusion is believed to be a source of VEGF drive, and the elevated VEGF levels may induce persistent macular edema [39].

### Assessing the extent of retinal non-perfusion

**Fig. 2:**
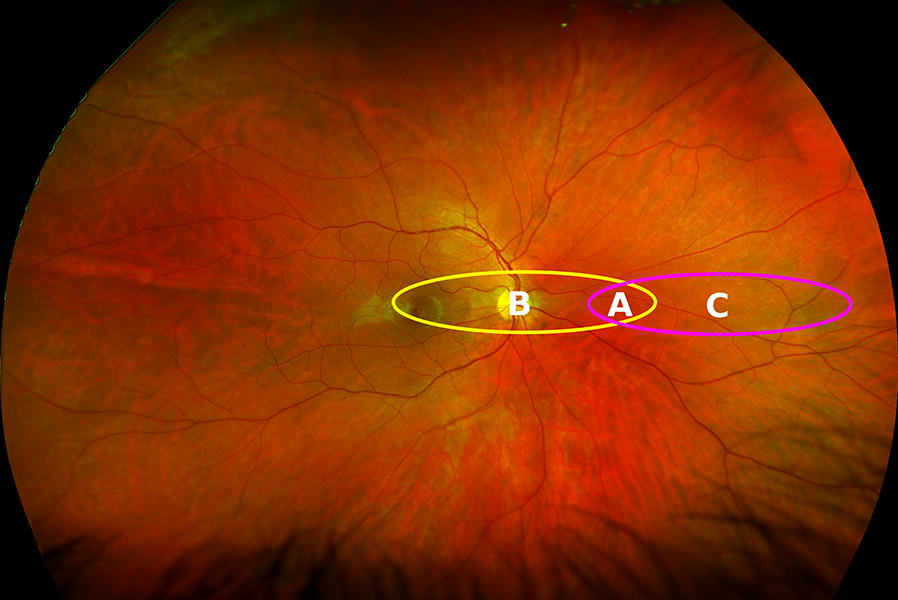
Ultra-widefield image of a human retina in stereographic projection where both annotated ellipses comprise 110,288 pixels. The area shared by both ellipses A is 3.59 mm^2^. The two ellipses labelled B and C have an area of 30.9 mm^2^ and 17.2 mm^2^ respectively (image courtesy of Jano Van Hemert)

In retinal imaging devices, the optics of a device in conjunction with the optics of the patient’s eye will map the 3-dimensional retina to a 2-dimensional image. This mapping distorts the image in a similar way when creating a flat map of the earth. For widefield imaging devices, this distortion will be larger than for traditional fundus cameras. By using stereographic projection, which mathematically projects from a three-dimensional structure to a two-dimensional image, directionality from a central point is preserved. Furthermore, this projection is conformal; it preserves angles where curves meet, which ensures shapes are not distorted. This property ensures that angles can be measured anywhere on the image, which is crucial for image registration between devices. While this projection may portray the relative locations of structures more accurately, it does so at the cost of equilaterality and equidistance, i.e., area and distance will not be the same throughout the image [33]. Because of this, lesions in the periphery appear larger than if they are located more centrally. As illustrated in Fig. 2, two ellipses of equal size on the image (each comprising 110,288 pixels) have areas of 30.9 mm^2^ and 17.2 mm^2^ respectively.

After images are represented in a stereographic projection, we can make accurate area, distance and angle measurements as the mathematics is widely known. The methods for making these measurements on retinal images were incorporated into the DICOM standard as Supplement 173 [44].

**Fig. 3:**
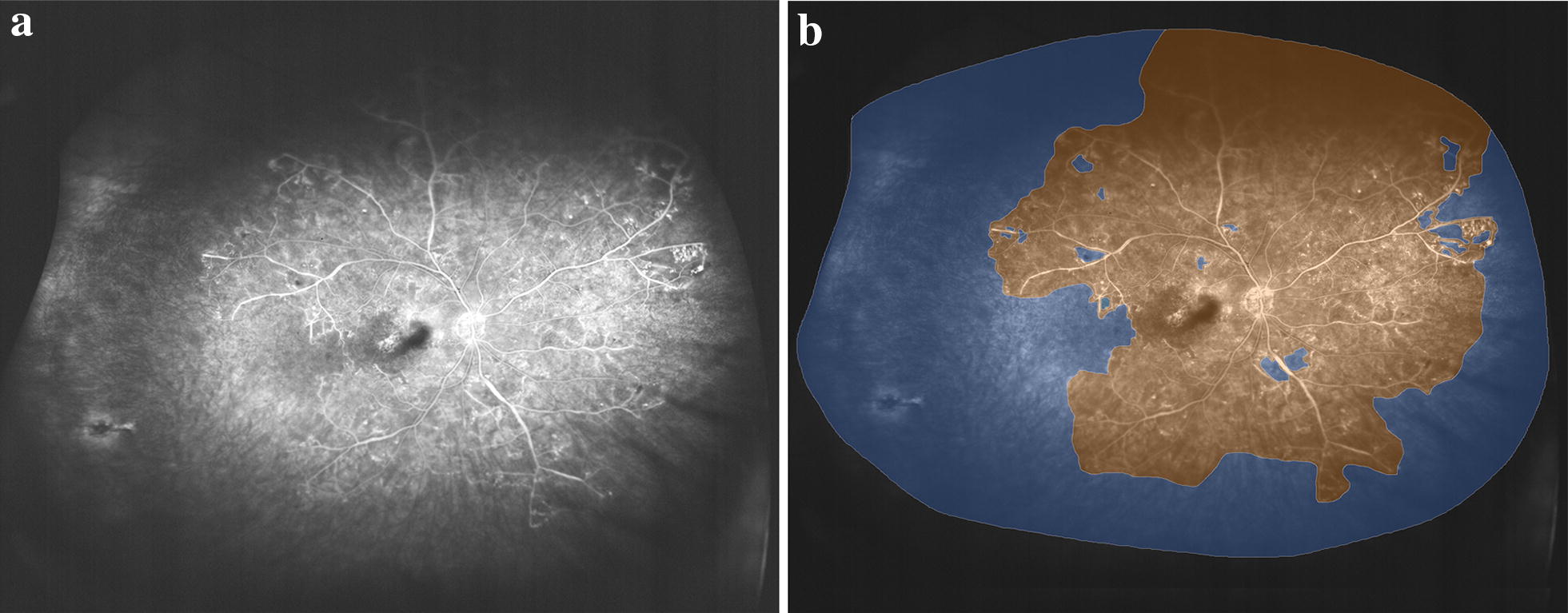
Ischemic central retinal vein occlusion. **a** Ultrawidefield fluorescein angiogram showing extensive areas of capillary non-perfusion throughout the posterior pole. **b** Grading diagram illustrating the regions of perfusion (orange) and ischemia (blue)

To facilitate assessment of areas of ischemia on UWF images, a method known as the ischemic index was described [45]. The ischemic index is the ratio of the number of pixels in the areas of non-perfusion to the total number of pixels of the visible retina (Fig. 3). Using the ischemic index, the amount of retinal non-perfusion in RVO was shown to vary considerably. Among patients with BRVO, the ischemic index varied from 0.1 to 61.3% [33, 39, 45]. The range of retinal non-perfusion in CRVO was even greater, varying from 0 to 99% [33, 39, 45].

The use of the ischemic index, however, has significant limitations. As mentioned earlier, lesions which appear to be similar in size may differ in actual area, depending on the location of the lesion on the image. As a result, the size of peripheral lesions may be overestimated, especially since ophthalmologists typically use reference structures in the posterior pole, such as the optic disc, to estimate the size of other structures in the fundus. Another consideration is that the area of visible retina varies considerably between eyes [33] and between the same eye at separate time points. Since the ischemic index is based on the ratio of non-perfusion to visible retina, variability in the area of visible retina could potentially confound the comparison of the extent of ischemia, and whether this is changing over time. Regions of the retina may also be difficult to grade because of artefacts caused by eyelids or eye lashes, especially in the periphery, or by media opacity such as vitreous hemorrhages. It is also uncertain whether the ischemic index obtained using different widefield imaging devices are comparable.

These limitations have been addressed by the use of stereoscopic projection software [33, 46, 47], which compensates for the device-specific three-dimensional (retina) to two-dimensional (an image of the retina) projection. In stereographic projection images, the same angle is maintained at every eccentricity. This means that angular distances are constant, and facilitates accurate measurement between points at various eccentricities. The stereographic projection software is built into the image capture software of the current Optos imaging devices, and allows accurate and precise measurement of areas and distances anywhere on the image.

Using stereographic projection, the area of non-perfusion in a cohort of RVO patients ranged from 0 to 365.4 mm^2^, with a mean of 95.1 mm^2^ and median of 49.6 mm^2^ [33]. This is equivalent to a mean of 36.7 disc areas and a median of 19.1 disc areas. The largest area of retinal non-perfusion (365.4 mm^2^) in this cohort is equivalent to 141 disc areas. Not surprisingly, the area of non-perfusion was larger in patients with CRVO (mean 135.8 mm^2^, range 5.3–365.4) compared to BRVO (mean 67.3 mm^2^, range 0–224.3) (p = 0.045) [33]. In a study of 22 patients [48], peripheral non-perfusion was detected on widefield imaging even though 7 of these patients were initially classified as perfused using CVOS study criteria. The mean area of retinal non-perfusion was found to be 368.7 mm^2^.

It has been shown that the area of non-perfusion correlates well with the ischemic index (R = 0.978, p < 0.001) [33]. However, measurement of areas of ischemia in anatomically correct units confers significant advantages, since these are units that ophthalmologists can relate to and understand more instinctively.

The total area of retina visible similarly has a wide range. In one cohort of patients with RVO, the total area of visible retina ranged from 559.4 to 797.7 mm^2^, with a mean of 690.6 mm^2^ [33]. In another study of the retinal vasculature which was conducted among normal subjects, the mean area of the normal perfused retina was 977.0 mm^2^. This was reported to vary with age, but not gender [49].

The area of retinal non-perfusion has been reported to change over time. In the Rubeosis anti-VEGF (RAVE) trial [50], all eyes demonstrated extensive areas of retinal non-perfusion. Patients experienced a mean loss of 8.1% of perfused retinal area per year, which is equivalent to 15 disc areas. The increase in retinal non-perfusion was 16.3% in the first year, 4.2% in the 2nd year and 3.6% in the third year.

Recently, the accuracy of apparent changes in non-perfusion over time on UWF FA has been questioned. Gaudric and colleagues, noted that in some regions of apparent recovered perfusion following anti-VEGF therapy, companion optical coherence tomography (OCT) angiography of these same regions appeared to suggest persistent capillary drop-out (presented at the International Retinal Imaging Symposium, Feb 2018, Los Angeles, California). The explanation for the apparent discrepancy remains uncertain and is a topic of active investigation, but would suggest caution in the interpretation of regions of suspected re-perfusion on FA.

## Impact of retinal non-perfusion on disease manifestations

### Retina and iris neovascularization

The extent of retinal non-perfusion appears to correlate with the likelihood of neovascularization. In one study, the mean ischemic index among 15 eyes with neovascularization was 75% (range 47–100%) whereas the eyes without neovascularization had a mean ischemic index of only 6% (range 0–43%) [45]. Of note, all eyes that had neovascularization had an ischemic index greater than 45%. The authors also found that the ischemic index had a significant correlation with presence of neovascularization.

### Relationship to macular edema

Studies have suggested that the extent and location of retinal non-perfusion may be related to the presence of macular edema in RVO. It is believed that up-regulation of VEGF production from the regions of non-perfusion may contribute to the development and severity of macular edema.

Prasad et al. [51] reported that non-perfusion anterior to the equator was strongly associated with macular edema in retinal vein occlusion. In contrast, non-perfusion that was isolated to regions posterior to the equator were not significantly associated with macular edema.

**Fig. 4:**
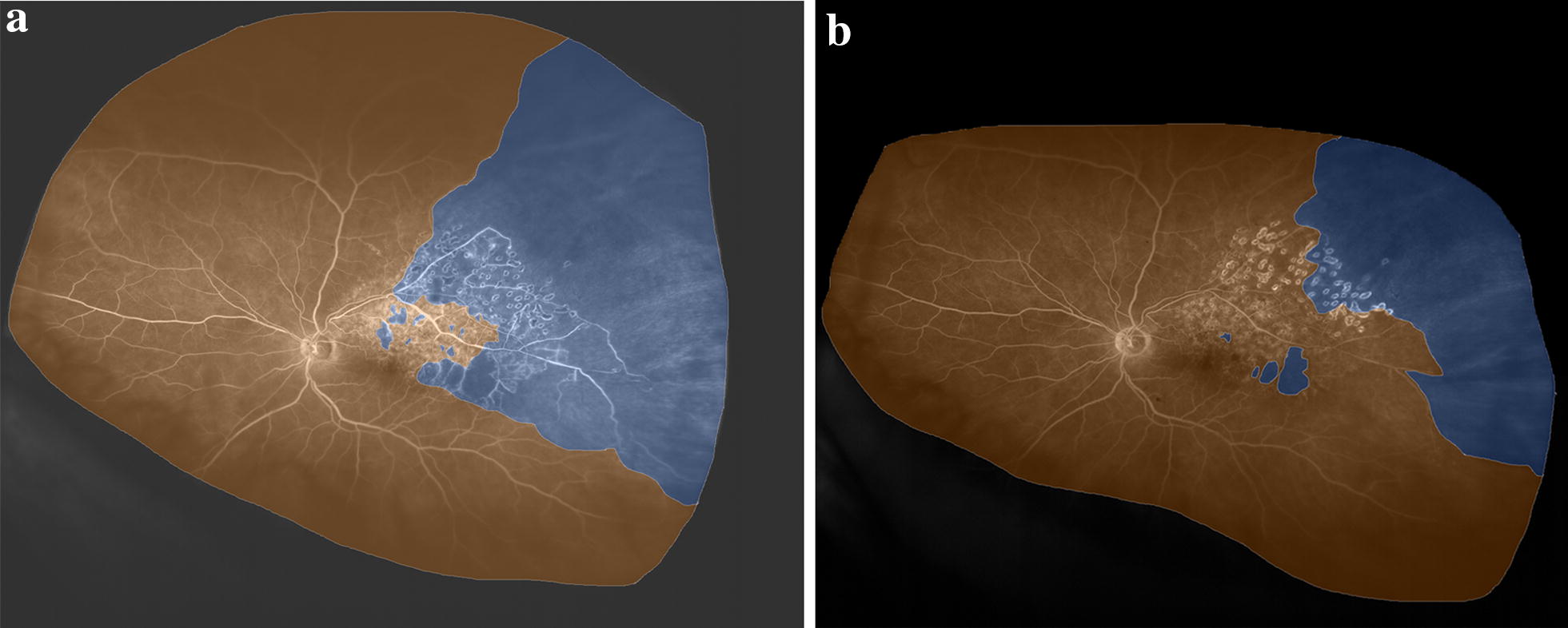
Branch retinal vein occlusion. **a** Widefield fluorescein angiogram (FA) before treatment, showing a large area of non-perfusion superotemporally (blue). Regions of perfused retina are shaded orange. **b** Widefield FA performed after treatment. The area of retinal non-perfusion has reduced

In a study of 32 patients with CRVO or BRVO, the mean ischemic index was higher when macular edema was present compared to when the edema had resolved following treatment (14.8% vs. 10.3%, p < 0.001) (Fig. 4). When subdivided by CRVO (13 patients) and BRVO (19 patients), similar trends were observed (22.5% when edema was present vs. 16.1% when edema had resolved for CRVO, p = 0.003; and 11.0% vs. 8.5% for BRVO, p = 0.003) [39]. In this same study, patients with ischemic index > 10% had greater amount of retinal thickening compared to those with ischemic index ≤ 10% (central subfield thickness 520.8 µm vs. 424.5 µm, p = 0.029).

In another study of 33 patients with BRVO, baseline central subfield thickness was 564 µm among those with between 50 and 100 fields of peripheral retinal non-perfusion compared to 373 µm among those with 0–49 fields of non-perfusion [52].

Investigators have suggested that the different regions of retinal ischemia may contribute to macula edema to different extents. In the WAVE study [53], which involved 24 patients with RVO, ischemic index was computed for the entire retina, as well for specific regions defined by a standardized grid: the perimacular area (PMA), near-peripheral area (NPA), mid-peripheral area (MPA) and far-peripheral area (FPA). All regions exhibited reduction in ischemic index compared to baseline. In this study, a significant correlation was found between central macular thickness and global ischemic index during follow-up (r = 0.22, p = 0.03). When different zones were analyzed, the PMA also showed a correlation with central macular thickness (r = 0.27, p = 0.007). The change in ischemic index was also correlated with changes in central macular thickness in the total retina, PMA and NPA.

## Impact of retinal non-perfusion on treatment and follow up

### Visual acuity

The extent of retinal non-perfusion may correlate with the final visual acuity in RVO. In a study of 53 patients with BRVO, patients with greater retinal non-perfusion (50 to > 100 fields) had final best-corrected visual acuity (BCVA) of 34.76 ETDRS letters, whereas those with smaller areas of retinal non-perfusion had BCVA of 40.63 letters [52].

In a study of 32 patients with CRVO and BRVO, those with > 10% ischemic index at baseline experienced a significantly larger gain in BCVA compared to those with ischemic index ≤ 10% (12.4 vs. 0.9 letters, p = 0.036) [39].

### Reduction in retinal edema

Changes in retinal thickness has been shown to vary according to the extent of retinal perfusion. Among patients with ischemic index > 10%, the mean decrease in OCT thickness was 296.1 µm compared to 165.3 µm for those with smaller areas of retinal non-perfusion [39]. A study of BRVO patients by Aghdam et al. [52] reported that central subfield thickness decreased by 222 µm (from 564 to 342 µm) among those with 50 or more fields of non-perfusion compared to 79 µm (from 373 to 294 µm) among those with less than 50 fields of non-perfusion.

### Number of anti-VEGF injections

Some authors have reported that the number of anti-VEGF injections varies with the extent of retinal non-perfusion. In a series of 54 patients with CRVO, patients with < 5 disc areas of non-perfusion had a mean of 4 injections, compared to a mean of 9 for those with > 5 disc areas [54].

In contrast, in a study of 32 patients with CRVO or BRVO, there were no significant differences in the number of anti-VEGF or dexamethasone implants administered between the groups with larger or smaller amounts of retinal non-perfusion [39].

### Targeted retinal photocoagulation

The observations described above have led some ophthalmologists to suggest a role for targeted retinal photocoagulation (TRP), where retinal photocoagulation is applied selectively to areas of ischemia seen on widefield FA. Since it is believed that the areas of ischemia are the source of VEGF drive, selectively photocoagulating those areas should theoretically be sufficient to reduce the VEGF drive, and consequently have an impact on the extent macular edema. By extension, applying laser to regions of healthy or perfused retina would theoretically be unnecessary as these areas would not be expected to contribute significantly to VEGF levels in the eye.

The Combination of Ranibizumab and Laser (CoRaLa) study was a prospective, randomized, interventional Phase IIb clinical trial of 22 patients with non-ischemic CRVO [55]. In this 6-month study, patients were randomized to receive intravitreal ranibizumab versus ranibizumab plus selective laser photocoagulation to areas of peripheral non-perfusion. The study showed that patients receiving combination therapy had better gains in BCVA (5 ETDRS letters vs. 0), and a smaller increase in areas of retinal non-perfusion compared to the monotherapy group.

Other studies have similarly reported improvements in BCVA [55, 56], reduction in macular edema [56, 57] and reduction in the number of anti-VEGF injections required [56] following the application of TRP.

In contrast, the Role of Laser in the Management of Retinal Vein Occlusion (RELATE) study reported no significant difference in BCVA between the group treated with monotherapy and combination therapy [58]. In the Wide-field Angiography Guided Targeted Retinal Photocoagulation Combined with Anti-VEGF Intravitreal Injections for the Treatment of Ischemic Retinal Vein Occlusion (WAVE) study [59], the proportion of patients gaining ≥ 15 letters was similar in both groups (33% vs. 38%), and the reduction in central retinal thickness was also similar (− 186 um vs. − 188 um (p = 0.99). Both groups also had similar number of injections (mean 9.5 vs. 8.8).

## Conclusion

UWF imaging plays an important role in the assessment and management of various retinal conditions. In RVO, UWF FA is essential for measuring areas of retinal non-perfusion, especially in the periphery. The extent of retinal non-perfusion may correlate with disease severity and treatment response.

## Abbreviations

RVO: retinal vein occlusion
CRVO: central retinal vein occlusion
BRVO: branch retinal vein occlusion
VA: visual acuity
VEGF: vascular endothelial growth factor
PEDF: pigment epithelium-derived factor
ETDRS: Early Treatment Diabetic Retinopathy Study
7SF: 7 standard field
UWF: ultra-widefield
CVOS: Central Vein Occlusion Study
RAVE: Rubeosis anti-VEGF
FA: fluorescein angiography
OCT: optical coherence tomography
WAVE: Wide-field Angiography Guided Targeted Retinal Photocoagulation Combined with Anti-VEGF Intravitreal Injections for the Treatment of Ischemic Retinal Vein Occlusion
PMA: perimacular area
NPA: near-peripheral area
MPA: mid-peripheral area
FPA: far-peripheral area
TRP: targeted retinal photocoagulation
CoRaLa: Combination of Ranibizumab and Laser
RELATE: Role of Laser in the Management of Retinal Vein Occlusion
